# Expression of *Vitis amurensis VaERF20* in *Arabidopsis thaliana* Improves Resistance to *Botrytis cinerea* and *Pseudomonas syringae* pv. Tomato DC3000

**DOI:** 10.3390/ijms19030696

**Published:** 2018-03-01

**Authors:** Mengnan Wang, Yanxun Zhu, Rui Han, Wuchen Yin, Chunlei Guo, Zhi Li, Xiping Wang

**Affiliations:** 1 State Key Laboratory of Crop Stress Biology in Arid Areas, College of Horticulture, Northwest A&F University, Yangling 712100, China; wmn_n@nwsuaf.edu.cn (M.W.); yanxun.zhu@nwsuaf.edu.cn (Y.Z.); hanrui1@nwsuaf.edu.cn (R.H.); yinwuchen123@nwsuaf.edu.cn (W.Y.); 2014060088@nwsuaf.edu.cn (C.G.); lizhi@nwsuaf.edu.cn (Z.L.); 2 Key Laboratory of Horticultural Plant Biology and Germplasm Innovation in Northwest China, Ministry of Agriculture, Yangling 712100, China

**Keywords:** *VaERF20*, grapevine, *Botrytis cinerea*, *Pseudomonas syringae* pv. tomato DC3000

## Abstract

Ethylene response factor (ERF) transcription factors play important roles in regulating immune responses in plants. In our study, we characterized a member of the ERF transcription factor family, *VaERF20*, from the Chinese wild *Vitis* genotype, *V. amurensis* Rupr “Shuangyou”. Phylogenetic analysis indicated that *VaERF20* belongs to group IXc of the ERF family, in which many members are known to contribute to fighting pathogen infection. Consistent with this, expression of *VaERF20* was induced by treatment with the necrotrophic fungal pathogen *Botrytis cinerea (B. cinerea*) in “Shuangyou” and *V. vinifera* “Red Globe”. *Arabidopsis thaliana* plants over-expressing *VaERF20* displayed enhanced resistance to *B. cinerea* and the bacterium *Pseudomonas syringae* pv. tomato (*Pst*) DC3000. Patterns of pathogen-induced reactive oxygen species (ROS) accumulation were entirely distinct in *B. cinerea* and *Pst*DC3000 inoculated plants. Examples of both salicylic acid (SA) and jasmonic acid/ethylene (JA/ET) responsive defense genes were up-regulated after *B. cinerea* and *Pst*DC3000 inoculation of the *VaERF20*-overexpressing transgenic *A. thaliana* plants. Evidence of pattern-triggered immunity (PTI), callose accumulation and stomatal defense, together with increased expression of PTI genes, was also greater in the transgenic lines. These data indicate that *VaERF20* participates in various signal transduction pathways and acts as an inducer of immune responses.

## 1. Introduction

In order to protect themselves from a wide variety of pathogens, plants have evolved an innate immunity system, comprising two inter-connected components: pathogen/microbe-associated molecular pattern (PAMP or MAMP)-triggered immunity (PTI) and effector-triggered immunity (ETI) [1,2]. PTI and ETI play important roles in defense networks, which are mediated by kinases and the production of pathogenesis-related (PR) proteins [2,3,4]. ETI is often accompanied by programmed cell death (PCD), also called the hypersensitive response (HR), which reduces the spread of an invading pathogen [2]. Moreover, ETI can also take part in an active defense response, such as the production of reactive oxygen species [5,6]. *Botrytis cinerea*, a kind of necrotrophic fungal pathogen, and the causal agent of grey mold disease, can produce the ROS in its plant hosts [7]. Also, ROS play an important role in the interaction between *Botrytis cinerea* and its plant hosts [8,9].

After infection by a pathogen, plants can activate hormone-mediated signaling pathways to defend against disease and various defense networks are known to be regulated by the phytohormones salicylic acid, jasmonic acid, and ethylene [10]. In *Arabidopsis thaliana*, the JA/ET signaling pathway plays an important role in resistance to necrotrophic pathogens, while the SA signaling pathway is thought to be necessary for mediating resistance to biotrophic pathogens, including the bacterium *Pseudomonas syringae* [11,12]. These different signaling pathways are associated with pathogen-induced transcriptional reprogramming. For instance, in *A. thaliana*, after inoculation with *B. cinerea*, a large number of genes show altered expression levels, including many transcription factors (TFs), such as members of the WRKY [13], NAC (*N*-acetyl cysteine) [14] and AP2/ERF (Apetala2/ethylene responsive factor) families [15]. 

The AP2/ERF superfamily is a large TF gene family, members of which are defined by a highly conserved 60–70 amino acid AP2/ERF DNA-binding domain, and is divided into ERF, AP2 and RAV sub-families, according to the number of AP2 domains they contain [16]. Proteins in the AP2/ERF superfamily with a single AP2 domain belong to the ERF sub-family, which have been shown to exhibit high DNA-binding activity [17]. Some ERF proteins bind particularly strongly to a GCC-box (AGCCGCC), a core DNA sequence in the ethylene-responsive element (ERE), which plays an important role in the regulation of plant responses to biotic stresses, especially pathogens [18]. In contrast, other ERF proteins can bind to the DRE/CRT (dehydration-responsive element/C-repeat element) *cis*-element and take part in abiotic stress responses [19]. Moreover, ERF proteins are involved in regulating SA/ET-inducible and pathogenesis-related (PR) gene expression [20,21] and mediating hormone defense networks, indicating that the ERF proteins are necessary for plants to respond to both biotic and abiotic stresses [22].

In previous studies, the ERF genes were shown to respond to abiotic stresses. For example, *A. thaliana CBF1* (C-repeat DRE Binding Factor 1), which binds to the DRE/CRT *cis*-element, was shown to be a transcriptional activator in response to low temperature and water deficit [23]. Similarly, cotton (*Gossypium hirsutum* L.) *GhERF38*, which is homologous to *A. thaliana AtERF38*, was up-regulated by drought, salt and abscisic acid (ABA) treatments [24]. Recent studies have also suggested that ERF proteins participate in plant growth and development. As an example, HL6, an AP2/ERF TF with only one AP2 domain, regulates trichome formation in rice (*Oryza sativa*) [25]. ERF proteins may also play a role in secondary metabolism, as the *Panax notoginseng* protein, PnERF1, binds to the GCC box *cis*-element involved in triterpenoid saponin biosynthesis [26].

ERF TFs have been shown to regulate immune responses in plants. *A. thaliana AtERF94* (*ORA59*), which belongs to ERF family group IX [27], was shown to act in the JA and ET signaling pathways and in defense mechanisms. Furthermore, overexpression of *ORA59* in *Arabidopsis* caused increased resistance to *B. cinerea* [28]. The expression levels of another gene belonging to group IX, named *AtERF1*, is induced by necrotrophic fungi such as *B. cinerea* and *Plectosphaerella cucumerina*, but remains unchanged after infection with *P. syringae* pv. tomato DC3000 (*Pst*DC3000), suggesting that the ET and SA signaling pathways play different roles in response to different pathogens [29]. In addition, some ERF genes, such as *AtERF078* (*AtERF4*) and *AtERF080* (*AtERF9*) from group VIII, have been shown to act as suppressors of resistance to *B. cinerea* [22,30], and in tomato (*Solanum lycopersicum*), pathogen-induced *ERF68* was shown to regulate cell death [31]. Studies to date have examined ERF protein function in disease resistance in species such as barley (*Hordeum vulgare*) [32,33], cotton [34] and wheat (*Triticum aestivum*) [35,36]; however, other crop species have received relatively little attention in this regard. For example, the yield from grapevines is influenced by *B. cinerea,* especially in some highly susceptible cultivars like “Red Globe” [37]. So, we think ERF TFs in grapevines may also play an important role in resistance to *B. cinerea*.

We previously identified genes through RNA-Seq analysis that were differently expressed after inoculation with *B. cinerea* in a comparison between the highly resistant *V. amurensis* Rupr “Shuangyou” and the susceptible *V. vinifera* cv ‘Red Globe’. In the current study, we cloned a grapevine ERF gene, *VaERF20*, which was chosen from the RNA-seq experiment, and over-expressed it in *A. thaliana* to examine its potential function in resistance to *B. cinerea* and *Pst*DC3000. We also evaluated the expression of SA, JA and ET related genes after inoculation by *B. cinerea* and *Pst*DC3000 to elucidate the function of *VaERF20*. Finally, we measured stomatal aperture and callose accumulation in epidermal peels following flagellin fragment (flg22) and lipopolysaccharides (LPS) treatments in order to obtain deeper insight into the role of *VaERF20* in bacterial resistance.

## 2. Results

### 2.1. B. cinerea Inoculation of Grape Induces VaERF20 Expression

**Figure 1:**
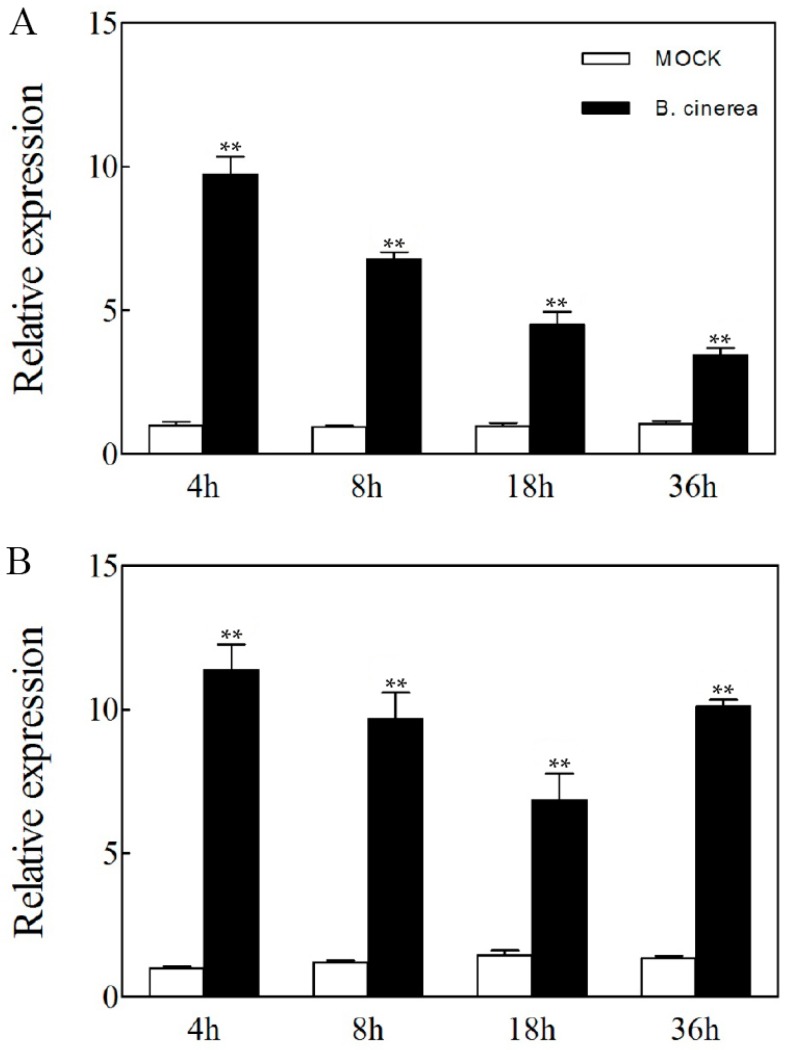
Expression analysis of *VaERF20* in Chinese wild *Vitis*, *V. amurensis* Rupr “Shuangyou” (**A**) and *V. vinifera* cv “Red Globe” (**B**) following inoculation with *Botrytis cinerea*. Data represent the means ± standard deviation from three independent experiments. Asterisks indicate statistical significance (** *p* < 0.01, one-way ANOVA) between treatment and mock.

To investigate the potential roles of *VaERF20* in responses to pathogen infection, we measured the expression levels of *VaERF20* in both the Chinese wild *Vitis* genotype, *V. amurensis* Rupr “Shuangyou” and *V. vinifera* cv “Red Globe” following inoculation with *B. cinerea* (Figure 1). *VaERF20* transcript levels peaked at 4 h post-inoculation (hpi) and increased in both genotypes after *B. cinerea* infection compared to the control.

### 2.2. VaERF20 Bioinformatic and Sequence Analysis

**Figure 2:**
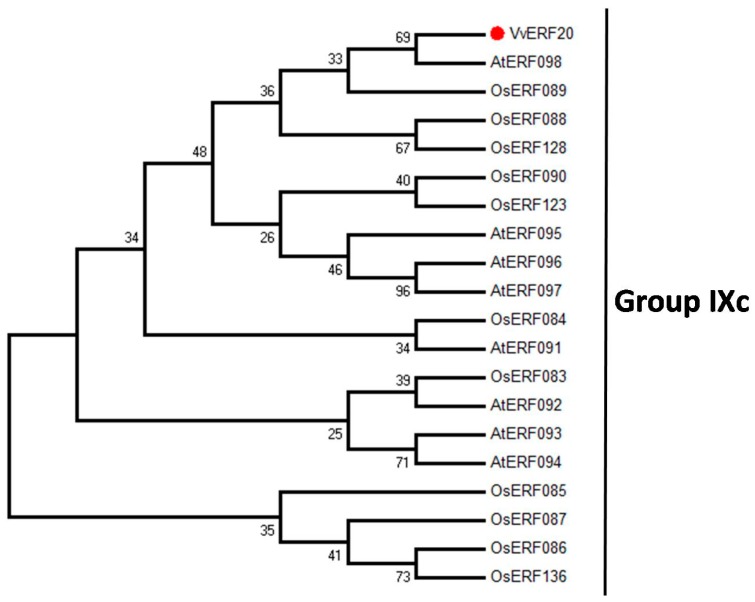
Phylogenetic analyses and multiple sequence alignment. Phylogenetic tree showing the relationship between *VaERF20* (red circle) and protein sequences from *A. thaliana* and *Oryza sativa*.

Phylogenetic analysis indicated that, of the protein sequences examined, *VaERF20* is most closely related to AtERF98 and OsERF89 (Figure 2), both of which belong to group IXc (B3) [27]. The importance of some genes from this group in the defense against pathogens has been shown [38]. The *VaERF20* nucleotide and deduced amino acid sequences are shown in Supplementary Data S1 and S2, with conserved domains highlighted by red arrows.

### 2.3. VaERF20 Transcript Profile When Over-Expressed in A. thaliana Lines

**Figure 3:**
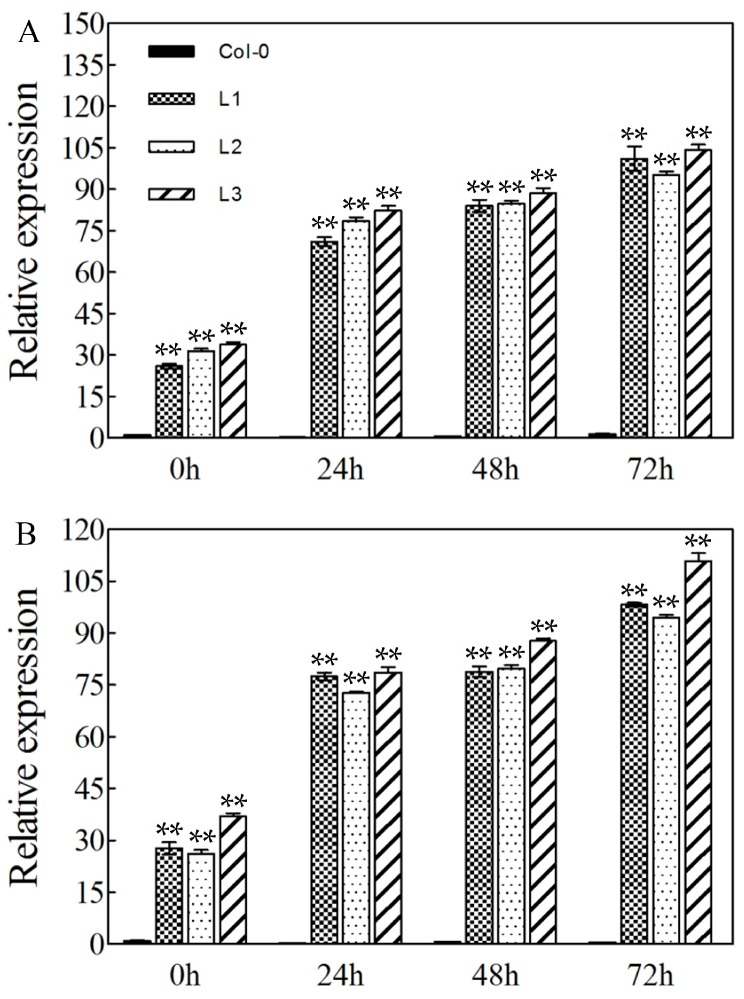
*VaERF20* expression analysis in three transgenic *A. thaliana* lines and Col-0 following inoculation with different pathogens. Quantitative real-time PCR for *VaERF20* expression patterns following *Botrytis cinerea* (**A**) and *Pst*DC3000 (**B**) inoculation. Data represent the means± standard deviation from three independent experiments. Asterisks indicate statistical significance (** *p* < 0.01, one-way ANOVA) between n transgenic *A. thaliana* and Col-0 samples.

The potential biological function of *VaERF20* in resistance to biotic stress was investigated by over-expressing it under the CaMV 35S promoter-driven in *A. thaliana* over-expressing plants. A total of 60 independent transgenic lines were generated and confirmed by PCR analysis. Three homozygous T3 lines with the strongest resistance to *B. cinerea* and *Pst*DC3000 infection (L1, L2 and L3) were selected for further study. The results of qRT-PCR analysis showing the expression levels *VaERF20* in the transgenic lines are shown in Figure 3, indicating that the transgene is expressed correctly in over-expressing lines. After *B. cinerea* and *Pst*DC3000 inoculation, the transcript levels of *VaERF20* in these three transgenic lines were 2.7–4.2 times higher than Col-0 at 0 hpi.

### 2.4. Overexpression of VaERF20 in Transgenic A. thaliana Increases Resistance to B. cinerea

**Figure 4:**
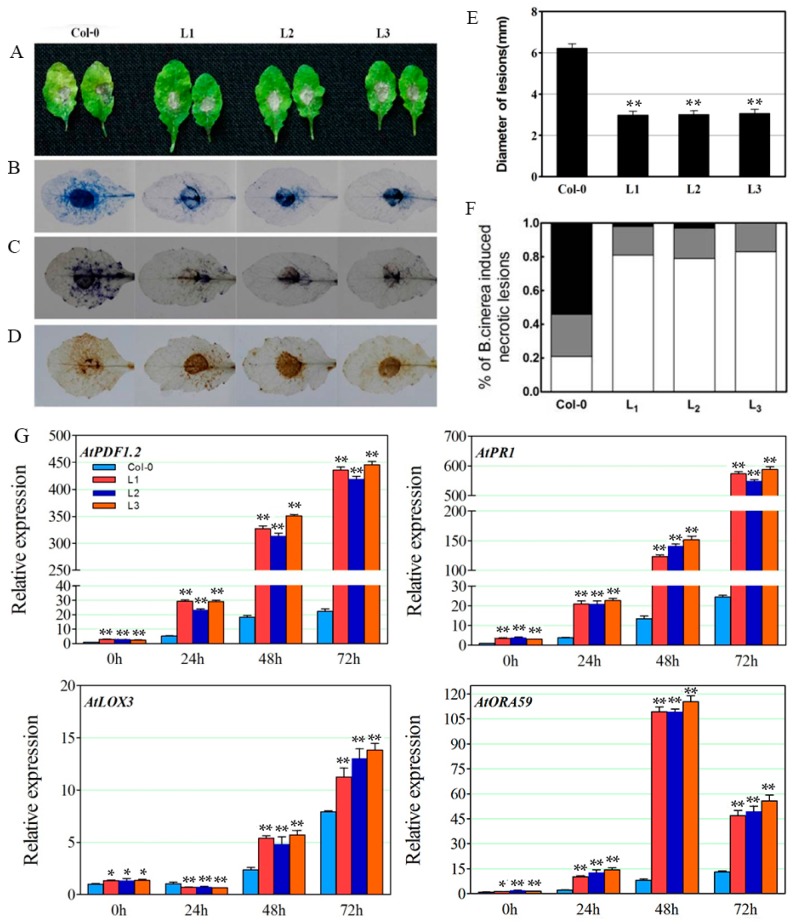
Overexpression of *VaERF20* in *A. thaliana* conferred enhanced disease response to *Botrytis cinerea* inoculation. (**A**) The disease symptoms on transgenic *A. thaliana* and Col-0 leaves three days post-inoculation. (**B**–**D**) Histochemical staining for cell death (**B**), O^2−^ (**C**) and H_2_O_2_ (**D**) accumulation, respectively. Transgenic *A. thaliana* and Col-0 were observed in three independent experiments with 5–10 leaves in each. (**E**) Average lesion diameter of *B. cinerea* at 3 days post inoculation (dpi). Data represent the means ± standard deviation from three independent experiments with at least 60 leaves per sample. Asterisks indicate statistical significance (** *p* < 0.01, one-way ANOVA) between transgenic *A. thaliana* and Col-0. (**F**) Symptoms on transgenic *A. thaliana* and Col-0 leaves at 3 dpi following *B. cinerea* inoculation by defining three lesion diameter sizes (<40% white parts, 40–80% grey parts, >80% black parts). (**G**) Expression analysis of defense-related genes in transgenic *A. thaliana* lines and Col-0 plants at 0, 24, 48 and 72 h post inoculation (hpi) with *B. cinerea*. Data represent the means ± standard deviation from three independent experiments. Asterisks indicate statistical significance (* 0.01 < *p* < 0.05, ** *p* < 0.01, one-way ANOVA) between *n* transgenic *A. thaliana* and Col-0 samples.

To examine the role of *VaERF20* in disease resistance, the three *VaERF20* over-expressing lines were inoculated with *B. cinerea*. In Col-0 wild type (WT) plants, typical *B. cinerea*-provoked lesions were seen at 3 dpi, while the *VaERF20* over-expressing *A. thaliana* lines exhibited no significant lesions (Figure 4A,E) and the proportion of large lesions were less than in Col-0 plants (Figure 4F). Histochemical staining revealed that the transgenic lines had higher levels of cell death (Figure 4B), O^2−^ (Figure 4C) and H_2_O_2_ (Figure 4D) accumulation than the WT plants.

Expression levels of defense-related genes were assessed by qPCR in response to *B. cinerea* inoculation. The expression of *PDF1.2*, which is involved in the JA/ET-mediated signaling pathway, showed a significant increase after infection (Figure 4G). In the transgenic plants, the expression was up-regulated by 20-fold compared to WT at 72 h after *B. cinerea* inoculation (Figure 4G). The expression of the pathogenesis-related gene 1 (*AtPR1*) and lipoxygenase-3 (*AtLOX3*), which play major roles in the SA-dependent disease resistance response and JA biosynthesis, respectively, also increased post infection (Figure 4G). *AtORA59*, which belongs to the ERF family and has previously been shown to respond transcriptionally to pathogen challenge, showed peak transcript levels at 48 hpi in the three OE lines (Figure 4G). The expression levels of *VaERF20* increased in the three transgenic lines following *B. cinerea* inoculation (Figure 3A).

### 2.5. Overexpression of VaERF20 in Transgenic A. thaliana Improves Resistance to Pst DC3000

**Figure 5:**
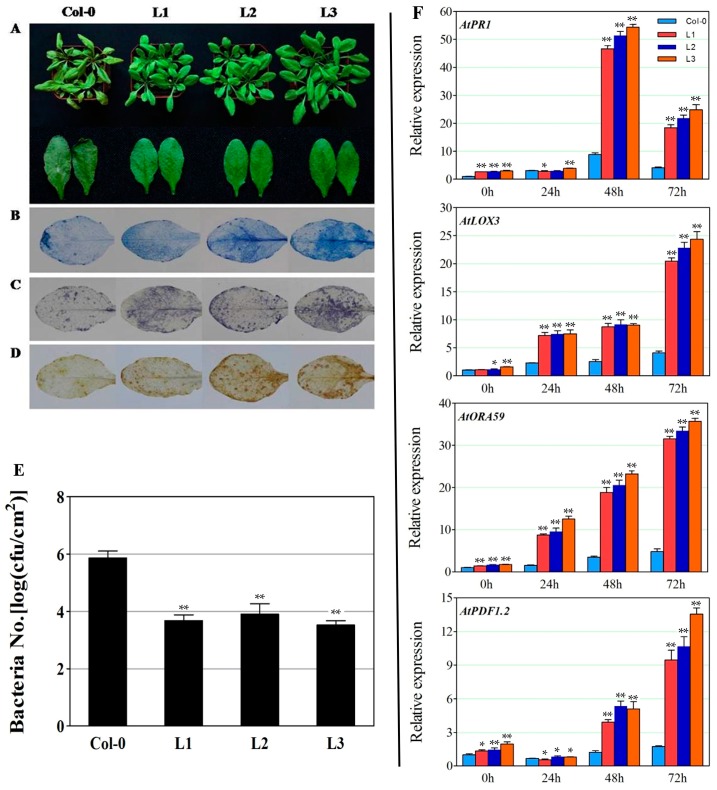
Overexpression of *VaERF20* in *A. thaliana* conferred enhanced disease responses to *Pst*DC3000 inoculation. (**A**) The disease symptoms on transgenic *A. thaliana* and Col-0 leaves three days post-inoculation (dpi). (**B**–**D**) Histochemical staining for cell death (**B**), O^2−^ (**C**) and H_2_O_2_ (**D**) accumulation, respectively. Transgenic *A. thaliana* and Col-0 were observed in three independent experiments with 5–10 leaves in each. (**E**) Bacterial population assays in infected transgenic *A. thaliana* and Col-0 leaves at 3 dpi. Data represent the means± standard deviation from three independent experiments. Asterisks indicate statistical significance (** *p* < 0.01, one-way ANOVA) between transgenic *A. thaliana* and Col-0. (**F**) Expression analysis of defense-related genes in transgenic *A. thaliana* lines and Col-0 plants at 0, 24, 48, 72 hpi following inoculation with *Pst*DC3000. Data represent the means ± standard deviation from three independent experiments. Asterisks indicate statistical significance (* 0.01 < *p* < 0.05, ** *p* < 0.01, one-way ANOVA) between n transgenic *A. thaliana* and Col-0 samples.

Three transgenic *A. thaliana* lines and Col-0 plants were inoculated with *Pst*DC3000 to elucidate whether *VaERF20* plays a role in bacterial resistance. At 3 dpi, disease symptoms were more severe in Col-0 plants, showing symptoms of chlorosis, while the three transgenic *A. thaliana* lines exhibited no such symptoms (Figure 5A). Accordingly, when bacterial populations were tested at 3 dpi, the quantities of bacteria in the three transgenic lines were lower than in Col-0 (Figure 5E). In contrast to *B. cinerea* infection, the frequency of cell death (Figure 5B) and the degree of O^2−^ (Figure 5C) and H_2_O_2_ (Figure 5D) accumulation were higher in the three transgenic *A. thaliana* lines compared to Col-0.

The expression levels of certain disease resistance related genes have also been shown to be affected within 72h of inoculation with this pathogen, and we therefore examined their expression profiles after *Pst*DC3000 inoculation. The expression of the *A. thaliana* JA signaling-related gene, *AtLOX3*, increased significantly by 72 hpi in the transgenic lines compared to Col-0 plants (Figure 5F). In a similar pattern to that resulting from *B. cinerea* infection, *AtPDF1.2*, *AtPR1* and *AtORA59* (Figure 5F) were all significantly up-regulated post-inoculation, while *AtPR1* expression peaked at 48 hpi and was ~6-fold higher in the transgenic lines than in Col-0. The expression of *AtPDF1.2* did not change at 24 hpi, but subsequently decreased and was still higher in the transgenic lines than in WT, while *AtORA59* expression rose gradually and peaked at 72 hpi. Finally, *VaERF20* expression levels increased more in the three transgenic lines than in Col-0 (Figure 3B).

### 2.6. B. cinerea Growth on Transgenic A. thaliana and Col-0 Plants

**Figure 6:**
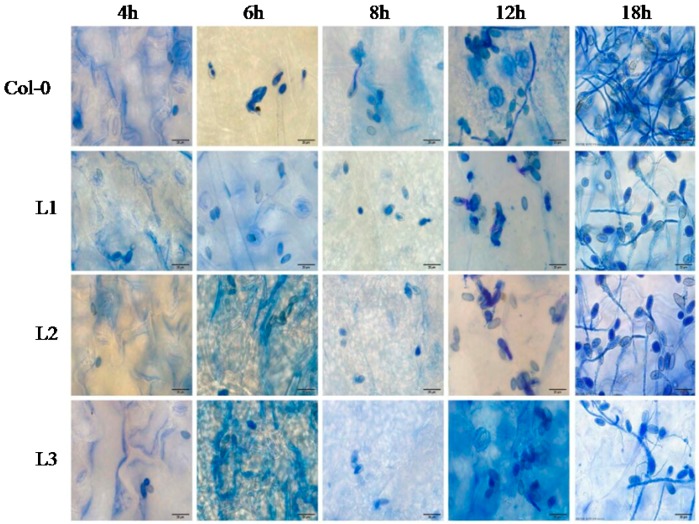
Comparison of *Botrytis cinerea* conidia development on transgenic *A. thaliana* and Col-0 leaves. Leaves were harvested at 4, 6, 8, 12, 18 h post inoculation (hpi) to detect progression of *B. cinerea* colonization and examined using a light microscope. The scale bar in the figure indicates 20 µm. Experiments were repeated three times.

We next characterized differences in growth of *B. cinerea* on the resistant transgenic lines and susceptible Col-0 plants (Figure 6). The degree of infection was more substantial in Col-0 and while no fungal growth was apparent at 4 hpi, germ tubes were detected on Col-0 leaves at 6 hpi. In contrast, almost no growth was observed in the transgenic lines, and *B. cinerea* conidia in the three transgenic lines germinated more slowly than in WT plants at 8 hpi. From 12 hpi, infection hyphae in Col-0 plants were also observed and their numbers increased until 18 hpi. Overall, hyphal growth was substantially perturbed in the transgenic lines compared to Col-0 at 18 hpi.

### 2.7. Overexpression of VaERF20 Causes Callose Accumulation in Transgenic A. thaliana in Response to Different Treatments

**Figure 7:**
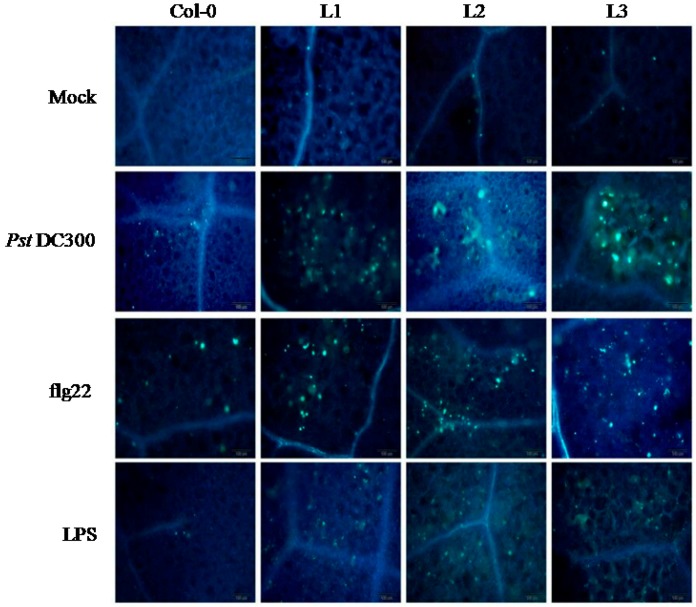
Callose deposition in transgenic *A. thaliana* and Col-0 leaves. Four-week old leaves were stained with aniline blue following inoculation with *Pst*DC3000, flg22 or LPS. Experiments were repeated three times with at least 5 leaves showing disease each repetition. The scale bar in the figure indicates 100 μm.

The cell wall polymer, callose, plays an important role in plant innate immunity at the early time-points of infection, as it can act as a physical barrier to repress pathogen elicitors [39]. When the DC3000 bacteria or the defense response elicitors flg22 and LPS were separately applied to the transgenic lines and Col-0, and the plants treated with the callose-binding stain aniline blue, we observed more callose in the transgenic lines than in WT (Figure 7).

### 2.8. Stomatal Closure Immunity Response

**Figure 8:**
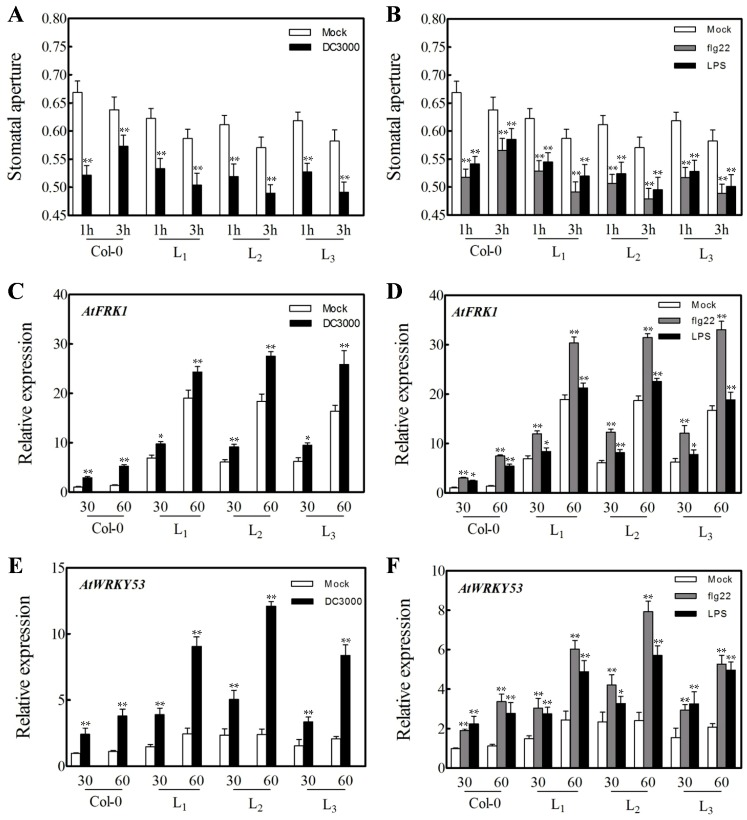
Response of stomatal apertures to *Pst*DC3000 infection, or flg22 and LPS treatment in transgenic *A. thaliana* and Col-0 leaves. (**A**, **B**) Statistical analysis of stomatal apertures (width/length; µm) following 1h and 3h of incubation. Data represent the means± standard deviation from three independent experiments with at least 60 stomata per sample. Asterisks indicate statistical significance (** *p* < 0.01, one-way ANOVA) between transgenic *A. thaliana* and Col-0. (**C**–**F**) Expression analysis of stomatal immunity response related genes in transgenic *A. thaliana* lines and Col-0 plants at 1h and 3h following inoculation with *Pst*DC3000, flg22 or LPS. Data represent the means ± standard deviation from three independent experiments. Asterisks indicate statistical significance (* 0.01 < *p* < 0.05, ** *p* < 0.01, one-way ANOVA) between transgenic *A. thaliana* lines and Col-0.

Stomatal closure is an important part of the induced plant innate immunity response [40]. We measured leaf stomatal aperture size in all three transgenic lines and Col-0 plants at 1 hpi and 3 hpi (Figure 8A,B). Stomata were predominantly closed in the transgenic lines and Col-0 at 1 hpi after *Pst*DC3000, flg22 or LPS treatments. However, stomata reopened in Col-0 plants during the third hour after incubation with DC3000, flg22 or LPS, while stomatal apertures in the transgenic lines still decreased.

To obtain a better understanding of the role of *VaERF20* in bacterial resistance, we measured the expression level of genes involved in this process. *FRK1*, a flg22-induced receptor kinase and the transcriptional regulator *WRKY53* both participate in PTI [41]. The expression levels of *AtFRK1* (Figure 8C,D) and *AtWRKY5*3 (Figure 8E,F) were both up-regulated after injection with *Pst*DC3000, flg22 and LPS compared to the mock controls, as was the expression of both genes compared to WT.

## 3. Discussion

The AP2/ERF gene family has previously been reported to be involved in the regulation of plant disease resistance pathways [18,29,42]. In this study, we cloned the *ERF20* gene from Chinese wild *V. amurensis* Rupr “Shuangyou”, and showed by sequence analysis that the protein has the conserved ERF domains (Supplementary Data S1 and S2) and share similarities with its counterpart in *V. vinifera* “Pinot Noir”. Phylogenetic analysis indicated that *VaERF20* belongs to ERF group IXc (B3). The B3 group plays a significant role in defense responses. One member, *ERF1*, enhanced disease resistance to *Fusarium oxysporum* in *A. thaliana* [29]. The *A. thaliana AtERF5*, *AtERF6*, *AtERF15* and *ORA59* genes play redundant roles in the defense against *B. cinerea* [18,22,28,43], while *AtERF15*, induces immunity against *Pst*DC3000 and *B. cinerea* [38]. We observed that overexpression of *VaERF20* in *A. thaliana* increased resistance to *B. cinerea* and *Pst*DC3000 compared to WT plants, and may also contribute to enhanced PTI responses after *Pst*DC3000, flg22 and LPS application. This suggests that it plays a role in the defense against both necrotrophic fungal and bacterial pathogens.

In pathogen-challenged plants, array recognition mechanisms and signal transduction defense-signal pathways are associated with a complex signaling cross-talk involving SA, JA and ET [44,45,46,47]. It has been proposed that the SA pathway is involved in resistance to biotrophic pathogens, such as *Pst*DC3000. Conversely, the JA and ET signaling pathways have been shown to be more effective against necrotrophic pathogens, such as *B. cinerea* and *F. oxysporum* [29,43,48,49]. Most studies have reported that the SA and JA/ET signaling pathways have mutually antagonistic interactions in regulating plant defense responses [46,50,51,52]. Interestingly, our data showed that the expression of both SA- and JA/ET-responsive defense genes was influenced after *B. cinerea* and *Pst*DC3000 inoculation. That conclusion is consistent with studies of *AtERF1* and *AtERF15*, which showed that these genes are induced by JA and ET as well as SA [38,53].

ROS have been shown to play an important role in plant responses to pathogen attack [5], and ROS accumulation is known to benefit *B. cinerea* infection [54] by facilitating programmed cell death and promoting infection, resulting in larger disease lesions [55,56]. Our results corresponded well with these findings. *VaERF20* transgenic lines showed enhanced resistance to *B. cinerea* compared to Col-0 plants, reflected in disease phenotype or conidial growth changes, and accumulated less ROS than did the Col-0 plants post-infection. This was different from *Pst*DC3000 inoculation, where the *VaERF20* over-expressing lines had increased resistance, but where the pathogen-induced ROS were higher in transgenic A. thaliana lines. The *Pst*DC3000 results correlate well with previous studies showing that pathogen-induced ROS increase immunity to this pathogen [57].

To obtain a better understanding of the effect of *VaERF20* over-expression in *A. thaliana*, we also examined the change in transgenic *A. thaliana* and Col-0 plants to defense response elicitors. Callose accumulation as a downstream signal of PTI showed diverse patterns in the two genotypes. Compared to WT, overexpression of *VaERF20* resulted in more callose accumulation post-inoculation with *Pst*DC3000. We observed that injection with flg22 and LPS also caused accumulation of more callose. A previous study has shown that callose can rapidly form a physical defense to protect cells from external injury [58]. Our result further suggests that *VaERF20* helps prevent pathogen spread. Another defense mechanism is stomatal defense, which has been shown to be important for PTI during bacterial challenge. We observed that the stomata of the transgenic *A. thalian*a plants remained closed for longer than in Col-0 plants, indicating that *VaERF20* over-expression caused an innate immunity response. We measured the expression of two PTI marker genes, *AtFRK1* and *AtWRKY53*, which were both fostered in transgenic *A. thaliana* more than in Col-0. These results not only further associate *VaERF20* with the innate immune response, but also support the hypothesis that *VaERF20* enhances defense against *B. cinerea* and *Pst*DC3000.

In conclusion, our data demonstrate that *ERF20* from Chinese wild *V. amurensis* Rupr “Shuangyou” can improve plant disease resistance. *VaERF20* over-expressing lines showed enhanced resistance to *B. cinerea* and *Pst*DC3000 infection and activated SA and JA/ET signaling. Our findings suggest that *VaERF20* is activated in plant responses to biotic stresses. Future studies will examine the transcriptional networks and regulatory mechanisms in which *VaERF20* operates in immune responses to pathogens.

## 4. Materials and Methods

### 4.1. Plant Materials and Growth Conditions

The Chinese wild *Vitis* genotype, *V. amurensis* Rupr “Shuangyou” and *V. vinifera* cv. “Red Globe” used for *B. cinerea* inoculation were obtained from the grape germplasm resources repository at the Northwest A&F University, Yangling, Shaanxi, China. *A. thaliana* ecotype Columbia-0 (Col-0) and transgenic lines were grown at 22 °C under long-day (16 h light/8 h dark) and 70% relative humidity conditions. All experiments used 4-week old *A. thaliana* and were repeated three times. Samples were immediately frozen in liquid nitrogen and stored at −80 °C until further use.

### 4.2. B. cinerea Inoculation of Grape

*B. cinerea* was isolated as previously described [37]. Briefly, conidia cultured on Potato Glucose Agar medium for 3 weeks were washed with distilled water. Conidia suspensions were pre-germinated for 2 h at 22 °C and a concentration of 105 spores/mL was used. Detached grape leaves were placed on 0.8% agar in trays and sprayed with *B. cinerea* conidia suspension. After inoculation, all leaves were incubated at 22 °C and a high humidity of 90–100% in the dark for the first 24 h and then in a light/dark (12/12 h) regime. Control leaves were sprayed with distilled water and incubated under the same conditions as the inoculated leaves. Samples were collected at 4, 8, 18 and 36 hpi (hours post-inoculation) for further analysis.

### 4.3. Generation and Characterization of Transgenic Lines

Total grape RNA from leaves infected with *B. cinerea* was extracted using the E.Z.N.A.^®^Plant RNA Kit (Omega Bio-tek, Norcross, GA, USA). First-strand cDNA was synthesized using the PrimeScript^TM^RTase 1st Strand cDNA Synthesis Kit (TaKaRa Biotechnology, Dalian, China) and diluted 6-fold with ultrapure water for subsequent analysis. The gene coding sequence (CDS) was obtained from the Grape Genome Browser (12×) (http://www.genoscope.cns.fr) and is also available through the National Center for Biotechnology Information (NCBI) (http://blast.ncbi.nlm.nih.gov/Blast.cgi) database. The *VaERF20* CDS was amplified by PCR using the gene-specific primers F (5′-GCTCTAGAATGGAAGGAGGAGGGAGGAGG-3′, *Xba*I site underlined) and R (5′-GGGGTACCCTAATTATTCCCTCTTCTATCACCCTGTG-3′, *Kpn*I site underlined), the PCR product was cloned into the pGEM-T Easy vector (Promega, Madison, WI, USA) and the sequence verified by sequencing. The CDS was then inserted in the plant expression vector, pCambia 2300, downstream of the CaMV 35S promoter (Cambia, Brisbane, QLD, Australia). 

The above construct was introduced into *Agrobacterium tumefaciens* (strain GV3101) using the floral dip method [59]. T0 seeds were harvested and grown on MS medium (pH 5.8, 10 g/L sucrose, 8 g/L agar) supplemented with 75 mg/L kanamycin. The T3 homozygous lines, L1, L2 and L3, which had the strongest resistance to *B. cinerea* and *Pst*DC3000, as well as high*VaERF20* expression, were selected from 60 independent lines.

### 4.4. Bioinformatic Analysis

The ERF protein sequences from rice and *A. thaliana* were as described in a previous report [27]. Phylogenetic analysis was performed using the MEGA 7.0 software and the neighbor joining (NJ) method [60,61] with 1000 bootstrap replicates.

### 4.5. Pathogen Inoculation Assays in A. thaliana

A *B. cinerea* spore concentration of 2.0 × 10^6^ mL^−1^ in sterile water with 4% maltose and 1% peptone [62] was used for inoculation assays. Ten ml conidia suspension was applied to 60 detached leaves from 25 four-week old transgenic and Col-0 *A. thaliana* plants and incubated at 22 °C under a 16 h light/8 h dark cycle with a humidity of 90–100% [63]. Lesion diameter and percentage of lesion areas were determined daily until 3 days. Samples were collected at 0, 24, 48, and 72 hpi for further use and experiments were repeated three times.

*P. syringae* pv. tomato DC3000 was cultivated at 28 °C in King’s B liquid medium overnight with shaking at 200 rpm [38]. The bacterial inoculum was re-suspended in 10 mM MgCl_2_ containing 0.02% Silwet L-77 to an OD600 of 0.02. Four-week old transgenic and Col-0 *A. thaliana* plants were soaked in DC3000 cell suspension for 10 min, and covered with a transparent plastic film to maintain a high humidity for 24 h [64]. To measure bacterial populations, leaf discs of inoculated leaves were rinsed with sterile water then homogenized in 100 μL 10 mM MgCl_2_, then the solution were gradually diluted and one hundred microliters of the resulting diluted solution were plated on KB agar plates as previously described [64]. Samples were collected at 0, 24, 48, and 72 hpi for further use and experiments were repeated three times.

To measure the stomatal response, 4-week old transgenic and Col-0 *A. thaliana* plants were exposed to 100 μmol·m^−2^·s^−1^ light for 3 h to ensure that 80% of the stomata were open. The epidermis of the leaves was immersed in 10 mM MgCl_2_, DC3000 suspension with OD = 0.02, 5 μM flg22 (Flagellin Fragment, Anaspec, Fremont, CA, USA) and 0.1 mg/mL LPS (lipopolysaccharides, Sigma, St. Louise, MO, USA) as previously described [63]. At least 30 fully expanded young leaves from 12 four-week old transgenic and Col-0 *A. thaliana* plants were peeled epidermis for each treatment. Transverse length and longitudinal width of stomata were measured using Image-J and samples were collected and observed under a microscope (Olympus BX51, Tokyo, Japan) at 1 and 3 h after treatment [63].

For the callose accumulation assay, 4-week old transgenic and Col-0 *A. thaliana* leaves were infiltrated with MgCl2, *Pst*DC3000, flg22 and LPS using a 1 mL needleless syringe, as described above. The injected leaves were examined for callose accumulation 18 hpi by aniline blue staining as previously described [65]. All experiments were repeated three times.

### 4.6. Histochemical Staining

Peroxide (H_2_O_2_), O^2−^ accumulation and cell death levels were carried out as previously described [65,66]. Briefly, H_2_O_2_ and O^2−^ accumulation was detected by 3, 3-diaminobenzidine (DAB) staining and nitro blue tetrazolium (NBT) staining, and cell death with trypan blue staining. The three histochemical staining methods were conducted with 30 detached leaves from 12 four-week old transgenic and Col-0 *A. thaliana* plants at 3 dpi [67]. One% (*w*/*v*) aniline blue (pH 9.5, dissolved in 150 mM K_2_HPO_4_) was used for visualizing callose deposition [63]. For observation of *B. cinerea* conidia development, leaves were collected at 4, 6, 8, 12, and 18 hpi, then decolorized in 3:1 (*v*/*v*) ethanol/trichloromethane containing 0.15% (*w*/*v*) trichloroacetic acid stored in 20% glycerol. Samples were then stored in 20% glycerol until examination with an Olympus BX-51 microscope by aniline blue staining [37]. All staining experiments were repeated three times.

### 4.7. Quantitative Real-Time PCR (qRT-PCR)

Total *A. thaliana* RNA from tissue inoculated with *B. cinerea* and DC3000 was extracted and first-strand cDNA was synthesized as described above. qRT-PCR was conducted using SYBR Green (TaKaRa Biotechnology, Dalian, China) according to the manufacturer’s instructions, using the following thermal profile: 95 °C for 30 s, 40 cycles of 95 °C for 5 s, and 60 °C for 30 s with StepOnePlusTM Real-Time PCR system (Applied Biosystems AB, Life Technologies Holdings Pte Ltd., Foster, CA, USA). Melt-curve analyses was performed using a program of 40 PCR cycles with 95 °C for 15 s, and then a constant increase from 60 °C to 95 °C. *VvActin* (GenBank accession number AY680701) or *AtActin1* (TAIR: AT2G37620) were used as reference genes. Specific primers used for qRT-PCR are listed in Supplementary Table S1. Relative gene expression levels were calculated using the 2^−^^ΔΔ*C*t^ method [38] and each reaction performed with three technical and three biological replicates.

### 4.8. Statistical Analysis

Data represent the means ± standard deviation from three independent experiments and were calculated using Microsoft Excel (Microsoft Corporation, Redmond, WA, USA). Statistical significance (one-way ANOVA) was determined using the SPSS Statistics 17.0 software package (IBM China Company Ltd., Beijing, China). All experiments were repeated in triplicate as independent analyses.

## Supplementary Materials

The following are available online at http://www.mdpi.com/1422-0067/19/3/696/s1.
