# Supplementary material for: Expression of Vitis amurensis VaERF20 in Arabidopsis thaliana Improves Resistance to Botrytis cinerea and Pseudomonas syringae pv. Tomato DC3000
Source: Int J Mol Sci. 2018 Mar 1;19(3):696. doi: 10.3390/ijms19030696 (PMC5877557; doi:10.3390/ijms19030696)
Supplement: Supplementary file 1 [file ijms-19-00696-s001.zip › Supplementary Table S1.pdf]

Table S1. Primers used for expression analysis of Arabidopsis thaliana resistance-related genes.

Gene	Gene locus TAG	Forward primer(5′to 3′)	Reverse primer (5′to 3′)
AtPR1	AT2G14610	AACTACGCTGCGAACACGTG	TCACTTTGGCACATCCGAGT
AtPDF1.2	AT5G44420	GAAGCACAGAAGTTGTGCGA	TGTAACAACAACGGGAAAATAAACA
AtORA59	AT1G06160	TTGTTATGACGCAAGAAAG	CCAACAGTAACGCAAATAG
AtWRKY53	AT4G23810	GCGACAAGACACCAGAGTCAA	CGCCGTTGATAGTTCCGTAA
AtFRK1	AT2G19190	GCCAACGGAGACATTAGAG	CCATAACGACCTGACTCAT
AtLOX3	AT2G35980	TCTCCGTACAACAAGCGTTGG	GCGTCCGTCTAGCGCATTAA
AtActin	AT2G37620	GGTAACATTGTGCTCAGTGGTGG	AACGACCTTAATCTTCATGCTGC
VaERF20	GSVIVT01028314001	CGGAGATACGAGACCCTTCAA	GAGAAAGTCTGCCCATACCCT
