# Supplementary figures and images for: Expression of Vitis amurensis VaERF20 in Arabidopsis thaliana Improves Resistance to Botrytis cinerea and Pseudomonas syringae pv. Tomato DC3000
Source: Int J Mol Sci. 2018 Mar 1;19(3):696. doi: 10.3390/ijms19030696 (PMC5877557; doi:10.3390/ijms19030696)

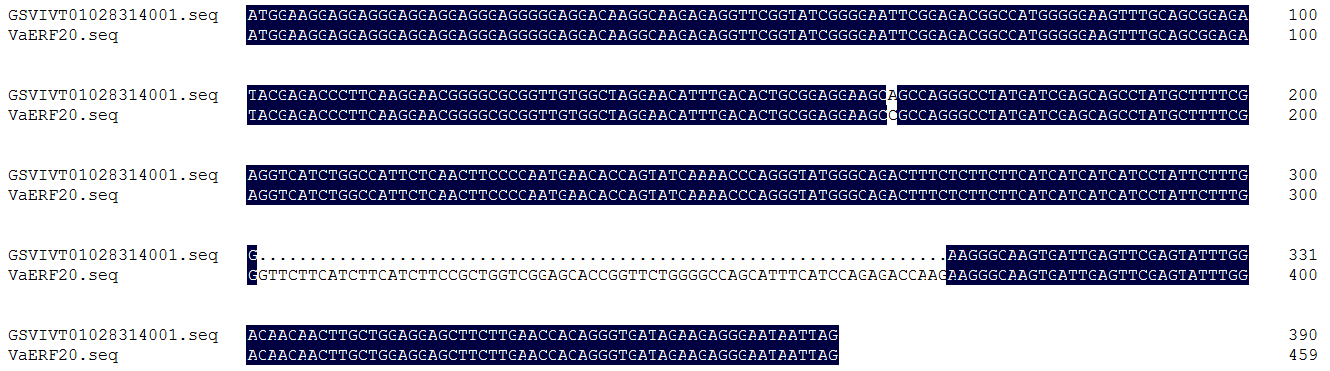

Supplement: Supplementary file 1 [file ijms-19-00696-s001.zip › Supplementary Data S1.jpg]

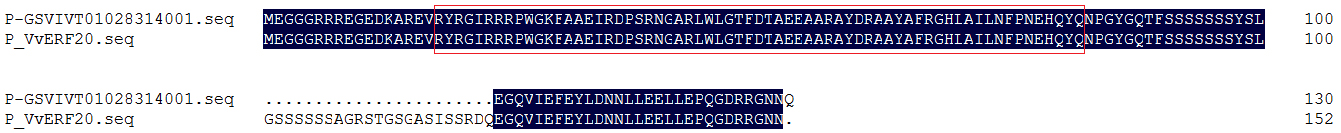

Supplement: Supplementary file 1 [file ijms-19-00696-s001.zip › Supplementary Data S2.jpg]
